# Feedback on Trunk Movements From an Electronic Game to Improve Postural Balance in People With Nonspecific Low Back Pain: Pilot Randomized Controlled Trial

**DOI:** 10.2196/31685

**Published:** 2022-06-10

**Authors:** Anita Meinke, Rick Peters, Ruud H Knols, Jaap Swanenburg, Walter Karlen

**Affiliations:** 1 Mobile Health Systems Lab Department of Health Sciences and Technology ETH Zurich Zurich Switzerland; 2 Department of Physiotherapy Occupational Therapy University Hospital Zurich Zurich Switzerland; 3 Directorate of Research and Education Physiotherapy Occupational Therapy Research Center University Hospital Zurich Zurich Switzerland; 4 Institute of Human Movement Sciences and Sport Department of Health Sciences and Technology ETH Zurich Zurich Switzerland; 5 Faculty of Medicine University of Zurich Zurich Switzerland; 6 Integrative Spinal Research Department of Chiropractic Medicine Balgrist University Hospital Zurich Switzerland; 7 Institute of Biomedical Engineering University of Ulm Ulm Germany

**Keywords:** low back pain, postural balance, exergame, postural feedback, motor control, kinesiophobia, inertial measurement unit, randomized controlled trial

## Abstract

**Background:**

Postural balance is compromised in people with low back pain, possibly by changes in motor control of the trunk. Augmenting exercising interventions with sensor-based feedback on trunk posture and movements might improve postural balance in people with low back pain.

**Objective:**

We hypothesized that exercising with feedback on trunk movements reduces sway in anterior-posterior direction during quiet standing in people with low back pain. Secondary outcomes were lumbar spine and hip movement assessed during box lift and waiter bow tasks, as well as participant-reported outcomes. Adherence to the exercising intervention was also examined.

**Methods:**

A randomized controlled trial was conducted with the intervention group receiving unsupervised home exercises with visual feedback using the Valedo Home, an exergame based on 2 inertial measurement units. The control group received no intervention. Outcomes were recorded by blinded staff during 4 visits (T1-T4) at University Hospital Zurich. The intervention group performed 9 sessions of 20 minutes in the 3 weeks between T2 and T3 and were instructed to exercise at their own convenience between T3 and T4. Postural balance was assessed on a force platform. Lumbar spine and hip angles were obtained from 3 inertial measurement units. The assessments included pain intensity, disability, quality of life, and fear of movement questionnaires.

**Results:**

A total of 32 participants with nonspecific low back pain completed the first assessment T1, and 27 (84%) participants were randomized at T2 (n=14, 52% control and n=13, 48% intervention). Intention-to-treat analysis revealed no significant difference in change in anterior-posterior sway direction during the intervention period with a specified schedule (T2-T3) between the groups (*W*=99; *P*=.36; *r*=0.07). None of the outcomes showed significant change in accordance with our hypotheses. The intervention group completed a median of 61% (55/90; range 2%-99%) of the exercises in the predefined training program. Adherence was higher in the first intervention period with a specified schedule.

**Conclusions:**

The intervention had no significant effect on postural balance or other outcomes, but the wide range of adherence and a limited sample size challenged the robustness of these conclusions. Future work should increase focus on improving adherence to digital interventions.

**Trial Registration:**

ClinicalTrials.gov NCT04364243; https://clinicaltrials.gov/ct2/show/NCT04364243

**International Registered Report Identifier (IRRID):**

RR2-10.2196/26982

## Introduction

### Background

Low back pain (LBP) contributed to most years lived with disability to the global burden of disease in 1990 as in 2017 [1]. The impact of LBP ranges from causing minor inconvenience to substantial restrictions in daily activities and, in extreme cases, disability and early retirement. Although there may be improvements when considering population age, the overall years lived with disability from LBP is rising and needs to be addressed [1]. Standard treatment recommendations for LBP often incorporate exercising and advice regarding physical activity [2], and it has been demonstrated that exercises for chronic LBP improve outcomes such as pain or disability to a certain degree [3-5]. Differences in effects between exercises with a distinct training focus have been appraised as negligible [4,5]. The limited effectiveness motivates the exploration of new ways for enhancing these treatments, as it has already been outlined by other authors [6]. Considering that changes of motor control of the lumbar region are discussed as a plausible cause for recurrence of LBP [7] and given that feedback plays a central role in motor learning [8], digital tools that make movement patterns more visible could be one such way to enhance exercise treatments. These interventions could be used together with other treatments as implemented in previous studies [6,9-11] or independently as needed, as a form of self-management. Many people with LBP do not request treatment, especially those with mild disability [12]. Therefore, supportive technology that can provide some degree of guidance at home, while maintaining independence, and could be especially interesting for this group of people.

Motor control can be described “...as the way in which the nervous system controls posture and movement to perform a specific motor task, and includes consideration of all the associated motor, sensory, and integrative processes” [7]. Physical characteristics and movement behaviors assessed to derive insights into deviations in people with LBP concerning these processes have revealed many new insights but still demand further clarification [7,13]. Examples of movement differences such as the limitations in range of motion (ROM) of the lumbar spine were found in all movement planes [14] and limited ROM of the trunk in the frontal plane but not in the sagittal plane seem to precede the occurrence of LBP [15]. Differences in the trunk region are thought to relate to differences in postural balance [7], which have been found by many studies [13,16,17]. Consequently, practicing movement tasks that focus on movement of the lumbar spine and hip could have the potential to enhance postural balance. Nevertheless, as highlighted earlier, the associations of LBP and movement behavior have not been fully clarified [7].

Altered movement behaviors seem to extend to tracing tasks, which work with feedback on trunk movements and have been used as an indicator for motor control in laboratory settings [18,19]. These studies used tasks that required participants tracing a circular pattern [18] or performing flexion movements [19] with their trunk, while receiving concurrent feedback. Results regarding the accuracy were conflicting, as one study found a difference between people with and without LBP in the accuracy of the tracing [18], whereas the other did not [19]. However, the latter study confirmed differences with respect to timing relative to the feedback between the groups [19]. Similar tasks may serve not only as a proxy measure of trunk motor control but also as training opportunity. It was recently found that practice to keep the lumbar spine constantly neutral during a box lift task was more successful when participants obtained digital feedback than when the participants used a mirror [20]. As described earlier, people with LBP seem to show movement patterns that deviate from those in people without LBP in different manners, such as displaying a high degree of rigidity or little control at all on their movement [7]. In tracing tasks, where movement becomes visible in relation to a target, people with LBP may develop well-coordinated trunk movements that are neither rigid nor loose. Furthermore, proprioception seems to be affected in people with LBP [21], which might relate to difficulties with fine-tuned trunk movements [18]. Early results indicate that there may be improvements in proprioception through interventions using feedback on trunk movement in people with LBP [22]. In short, we assume that such training may reduce postural balance impairments by restoring more adequate movement behaviors of the trunk.

The effects of exercising interventions directly on postural balance have been studied previously. Meta-analyses on intervention studies with older people suggest that balance training [23] and Pilates [24] but not programs focusing on strength or mixing different kinds of exercises [23] can enhance postural balance. Although these results originate from older participants, studies in people with LBP imply similar development of pain [25] and treatment results [26], largely independent of age. Furthermore, altered trunk characteristics have also been reported in younger people with LBP [27]. Multiple interventional studies with people with LBP found an impact of exercising interventions on at least one of the investigated criteria describing balance [28-30], whereas in another study no differences in postural balance were detected [31]. However, different tasks with varying requirements were used; for example, standing on moving ground [28], standing on a single leg [29], and assessments in squat positions [30].

Reviews so far revealed mixed success of digital tools for exercising [32] and encouraging early results for virtual reality applications [33] in people with LBP. The present literature has been described as heterogeneous with respect to the interventions [32] and methods [33]. Technical progress is considered as a factor to possibly impact intervention effectiveness, although so far, no clear difference for later studies was observed [32]. Thus, consistent research effort is necessary. Digital tools can be designed to influence the way movements are performed during exercises, for instance, to vary the speed [34]. Investigated tools include exergames readily available in the market; for example, exercising with the well-known Wii balance board (Nintendo of America, Inc) [35,36] or the Valedo Motion (Hocoma AG) [6,11]. Sensor technology has been used to intervene on movement characteristics of the trunk directly; for example, in studies [37,38] where warning participants from performing extreme back movements during everyday work was investigated. Other interventions have specifically encouraged movement of the lumbar spine in an exercising context [9,11,22,39] or are otherwise dedicated to providing feedback on lumbar spine movement [9-11,22]. For different kinds of tools used, only a small amount of research has been conducted [32]. Therefore, such digitally supported training modalities should further be investigated. Different systems and technological setups have been explored, for instance, cameras [40], wearable sensors [6,41], and sensors readily available in mobile phones [42], sometimes in combination with virtual reality headsets [41,42]. However, only few studies provide first insights in the effects of these interventions on movement quality in people with LBP [9-11]. In all, 2 studies suggested such interventions might have positively affected trunk ROM, but in one study, it remained ambiguous whether there was a significant difference to the standard care control group [9] and in the other study only a single group was investigated [22]. In a third study no effect on ROM was found [10]. Motor control impairment was not different in a study, where patients in the intervention group received access to additional exercises with sensor-based feedback other than the control group [11]. To our knowledge, the effect of such exercises on postural balance in people with LBP has not yet been investigated.

### Objectives

The primary aim of this study was to examine whether exercising with feedback on trunk movements can enhance postural balance, indicated by the change in anterior-posterior (AP) postural sway between the assessments before and after the intervention. Additional parameters to quantify postural balance were explored. As secondary outcomes, movement of the lumbar spine and hip during 2 different movement tasks and participant-reported outcomes were included. A further aim was to analyze adherence to the intervention.

## Methods

The completed CONSORT-EHEALTH (Consolidated Standards of Reporting Trials of Electronic and Mobile Health Applications and Online Telehealth) checklist [43] is provided in Multimedia Appendix 1 [2-12,20,22,32,39-42,44-50]. The intervention is described according to the TIDieR (Template for Intervention Description and Replication) checklist [51].

### Study Design

**Figure 1:**
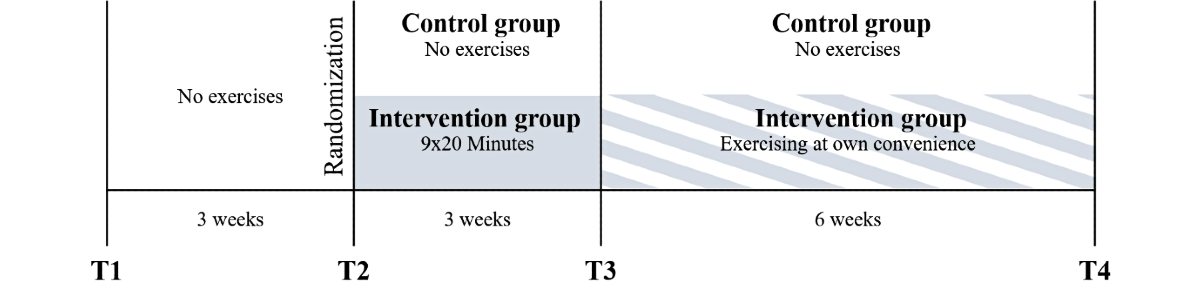
Overview of the study schedule showing assessment visits T1 to T4 and the interventions.

This manuscript was based on a study protocol [44] that included a 2-arm randomized controlled trial. Figure 1 shows the assessment and intervention schedule. The study took place at University Hospital Zurich between May 2019 and October 2020. Except for an extension of the study period of 3 Months to compensate for a pause due to the COVID-19 pandemic, the study was completed as planned, and interim analyses of intervention effects were not conducted. Outcomes were assessed twice at T1 and T2, before an intervention was given. Further assessments were taken after another 3-week period with a fixed exercising schedule for the intervention group (T3) and a subsequent 6-week period without specified exercising schedule (T4). Participants were randomized during the assessments at T2, and those assigned to the intervention group received an introduction to the exercising program right after the assessment. After T3, participants in the intervention group retained the Valedo Home exercising system (Hocoma AG), without being required to follow a specific schedule or to complete any exercises at all. This period was introduced to observe further adherence to the exercising program, without commitment to a schedule provided by a therapist or to complete a schedule for research purposes. Participants who were randomized to the control group did not receive a sham intervention.

Block randomization (blocks of 2 and 4), stratification by body height, and 1:1 allocation were implemented through the randomization tool in REDCap (Research Electronic Data Capture; Vanderbilt University) [45] hosted at Eidgenössische Technische Hochschule Zurich. AM generated random sequences with the dedicated R package blockrand (version 1.3; [49]) and randomized the participants using REDCap. The staff conducting assessments of the outcomes was blinded, and randomization occurred as late as possible (at T2) to reduce the risk of accidental unblinding.

The published study protocol [44] contained a further research question involving an additional patient group. We intended to compare the effect of the intervention between a group of patients and other participants who did not receive other treatments than the exercise intervention with postural feedback. In this manuscript, we report only the research questions that could be investigated based on the collected data, as insufficient patients were enrolled at the study site.

### Participant Recruitment

Participants were recruited through different web-based bulletin boards, websites, distribution of flyers, and personal communication. Recruitment was completed 3 months before the planned end date, to allow all participants to finish in time. Eligibility was ascertained in an interview-like setting that allowed the participants to describe their situation. Participants were considered eligible if they provided informed consent, they were aged at least 18 years, reported to the investigator that they had nonspecific LBP, and did not receive therapy or medical treatment for LBP within the past 6 months. There was no questionnaire-based assessment or formalized cutoff scores for pain intensity during the eligibility check. If it was not possible to clarify the eligibility of a participant by asking standard questions alone, one of the trained physiotherapists of the team decided on the criterion in question. The criterion of no recent treatment was relaxed from 12 months to 6 months during the study to improve recruitment rates. Participants reporting specific LBP or radicular syndrome were excluded from participation. Participants were also excluded if they indicated to the investigator that they would not be able to complete the movements required by the exercise intervention owing to high pain. Other reasons for exclusion were pregnancy, taking medication that impairs postural balance, severely impaired vision, allergic reactions to adhesive strips, and insufficient proficiency in German or English.

### Ethics Approval

Participants were not compensated and provided informed consent in writing before any study procedure was started. The trial was approved by the Cantonal Ethics Committee Zurich (BASEC: 2018-02132) and registered in ClinicalTrials.gov (NCT04364243).

### Outcomes and Procedures

#### Postural Balance

Records of center of pressure (COP) during quiet standing on a stable force platform (AMTI, Accusway Plus) were used to quantify postural balance. Specifications of the number of repetitions, duration, instructions, sampling rate, and filter cutoff frequency were based on relevant literature [52] and are described in detail in this section. During the assessment, the participants stood as quietly as they could, with the arms relaxed at the side and eyes closed, while wearing opaque goggles. Each participant selected an individually comfortable, *usual* foot position. To keep the stance consistent for each participant during all balance assessments, the foot position was recorded on a plastic foil. Participants wore socks but no shoes on the platform. A total of 4 postural balance trials of a duration of 120 seconds were recorded with a sampling rate of 100 Hz at each assessment visit. The data were filtered using a fourth order low-pass Butterworth filter with a cutoff frequency of 10 Hz. Data from the first and last 5 seconds were removed from the records to permit a stabilization phase at the beginning and to assure that any effects of a lateral leaning movement used for time synchronization with additional sensor data were removed with a safety margin. Thus, parameter estimates for each repetition were based on segments of 110 seconds. Mechanisms regulating balance in AP and medio-lateral direction differ [53]; therefore, directional measures were used to quantify balance in addition to measures irrespective of the direction on the 2D plane (global). The trajectory of the COP was described by the mean absolute displacement from the mean COP (AP, medio-lateral, and global), and by corresponding velocities, graphically represented in Multimedia Appendix 2 [54]. Change in displacement in AP direction (T3-T2) was a priori defined as the primary outcome. The data were reported on a mm and mm/s scale, and reduction in displacement and velocity were the favorable outcomes.

#### Movement Tasks

**Figure 2:**
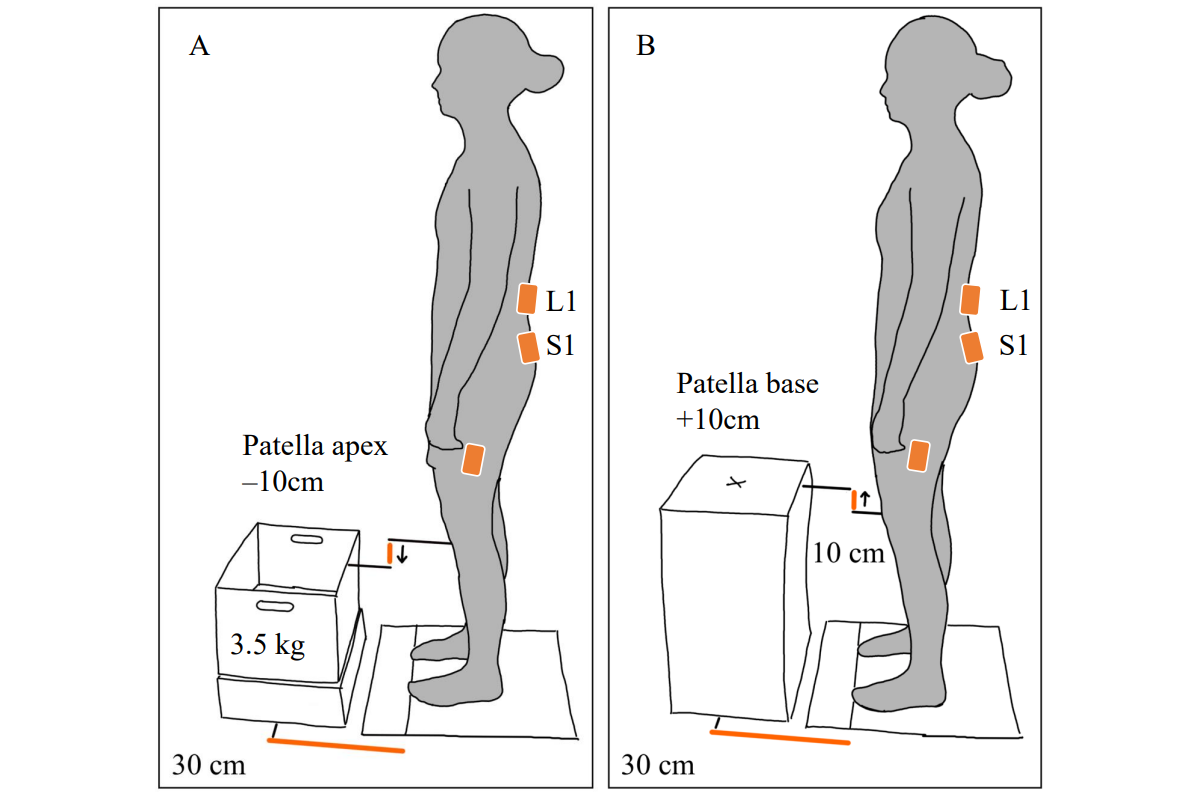
Setup of the movement tasks, inertial measurement unit positions (orange markers), and task material adaptation for A: box lift task and B: waiter bow task. The specifications have been adopted from Matheve et al [46].

Further assessments during movement tasks were performed to see whether the participants were able to follow the instruction to limit movement of the lumbar spine and perform movements on the sagittal plane by bending the hip joint instead. The protocol and setup of these assessments were adopted from the study by Matheve et al [46]. The assessments taken during box lift and waiter bow tasks were shown to reliably determine the change of the lumbar spine and hip posture during these tasks [46]. Similar versions of these tasks have been used elsewhere [20]. Figure 2 shows the setting and adaptation of the tasks to the individual participants. Lumbar spine and hip angles were used to describe the performance during these tasks. The Valedo Pro (Hocoma AG), consisting of 3 inertial measurement units (IMU) and dedicated software, was used for the assessments. The IMUs were placed with medical adhesive strips at the height of the spinal process of the S1 and L1 vertebrae, and 1 IMU was placed on the left leg, 20 cm from the lateral femoral condyle. Sensor positions were identified by palpation. Different from the aforementioned study [46], we did not alter the participants natural spinal posture before the tasks were performed. We assumed the tasks would otherwise be selectively more difficult to perform for participants who received more intense corrections to their posture. In addition, we allowed only 1 practice trial before the 5 repetitions of each task to keep learning effects minimal. By tracing the position of the feet to a foil, the position was standardized across assessments.

The box lift task required the participants to lift a box and hold it during upright standing and put the box down again and return to the standing position. For the waiter bow task, the participants were asked to touch a marked spot positioned in front of them with the fingers by bending from the hip joints and return to upright stance. The central instruction was to not change the alignment of the lumbar spine while performing the tasks. During the waiter bow task, participants were instructed to keep their knees at the original angle. The participants stood with their feet parallel, at a self-selected width, in a predefined distance to the task materials. Correct task execution and possible mistakes were shown to the participants by the outcome assessor. The order of tasks was randomized for each assessment visit. The data were collected at a sampling frequency of 50 Hz and change in lumbar spine angle was calculated by subtracting the rotation of the S1 sensor on the sagittal plane from the rotation of the L1 sensor on the sagittal plane. The obtained data were filtered using a moving average of 0.2 seconds, and the maximum absolute departure of the position at task beginning was used as the end point. Hip angles were obtained analogously, using data from the IMU at S1 and IMU at the thigh.

#### Participant-Reported Outcomes

Before the movement assessment at each visit, the participants completed a questionnaire in English or German, on a laptop. Considering the recommendations regarding relevant outcome assessments for studies on LBP [55], we included questionnaires covering pain intensity, disability associated with LBP, and quality of life (QOL). A 11-point numeric rating scale (NRS) asking participants to rate their pain intensity during the previous week, with the anchors *no pain* and *worst imaginable pain,* was applied additionally at the first assessment [56].

The Roland Morris disability questionnaire (RMDQ) was used to measure disability [57,58]. Respondents selected those of the 24 statements, which they experienced on the date of assessment, resulting in scores from 0 to 24 [57,58]. The RMDQ is an established questionnaire with adequate psychometric performance [59].

The World Health Organization Quality of Life Questionnaire-short version (WHOQOL-Bref) includes 26 items, which cover different aspects of QOL: physical health, psychological QOL, social relationships, and environmental factors [60]. The score of the physical health subscale is calculated by averaging the responses of 7 items (5 response options per item multiplied by 4) [60]. The selection of questions for the WHOQOL-Bref was based on data from international samples [60], and it was found to be reliable and valid [60,61].

The Tampa Scale for Kinesiophobia, 11- item version, was used to measure fear of movement, and the sum scores (4 response options: 11 to 44) were analyzed [62,63]. The English and German versions were found to generate reliable and valid data [62,63].

#### Baseline Characteristics and Adherence

The questionnaire at T1 contained questions regarding the participants age at the first occurrence of LBP, days with LBP during the previous month, and the average LBP intensity (*When you have back pain, how would you rate your average low back pain intensity in general?*), using labels of *no pain* and *worst imaginable pain* to describe the minimum and maximum values of 0 and 10, respectively. In addition, demographic data were collected. Weight and height were assessed at the study site. The exercises that were performed at home and the matching time stamps were extracted from the Valedo Home app.

### Intervention

The intervention is described in Textbox 1. Multimedia Appendix 3 contains a video on the exercises used in this study (published with permission from Hocoma AG).

**The TIDieR (Template for Intervention Description and Replication) checklist items [51] for exercising, with postural feedback.**

**Item and intervention description**

- Brief name: exercising with postural feedback on trunk movements using the Valedo Home system (Hocoma AG).
- Rationale: postural balance deficits in people with low back pain may stem from disturbed coordination of the trunk. We assume that practicing trunk movements with a feedback system helps participants to learn to control their trunk precisely. This improved control of the trunk could in turn affect how well balance can be controlled in people with low back pain.
- Materials: the Valedo Home system and belts or medical adhesive strips that were used for attaching the sensors to the chest and lower back. A tablet (Huawei Media Pad T5) with the Valedo app, a paper document summarizing the instructions, and the user manual [47] were provided.
- Procedures: participants randomized to the intervention group were instructed how to use the Valedo system and performed 1 exercise under supervision at T2. During this training session, the participants learned how to place the sensors correctly and to use the tablet and the Valedo app. At each of 9 home exercising sessions, the participants performed 10 exercises. Multimedia Appendix 3 contains a video showing all the exercises. The participants practiced moving their trunk and pelvis precisely to guide an avatar along a specified path with their movements through a virtual world. The exercises consist of movements of the upper body or the pelvis. Trunk movements are performed on the sagittal, frontal, and transversal plane, and hip movements are performed on the sagittal and frontal plane. Participants see on the display how well they match the specified movement trajectory while playing, and further auditory feedback is provided. At the end of the game, a ranking of the current and previous performance in the game is provided. After the assessment at T3, the participants in the intervention group were informed that they could keep using the system until T4 and that there was no specific schedule to complete, and they could use the system at their own convenience.
- Provider: the exercises were delivered by the Valedo Home system. AM trained the participants and acted as the contact person during the study. The participants were encouraged to contact AM, if any questions or technical difficulties should occur.
- Mode of delivery: each participant was instructed individually. Exercises were guided by the Valedo Home system.
- Location: instructions were provided at University Hospital Zurich, and the regular exercises were performed by the participants at home.
- Frequency and duration: the participants completed 10 exercises repeatedly, with an effective duration of 20 minutes, in 9 sessions until T3. Participants were told to space out the exercising sessions approximately equally between the appointments, but the exact dates were not defined. After T3, the participants could choose the exercises and duration by themselves.
- Tailoring: the exercises are adapted to the range of motion of the participant, which is measured as part of the user profile setup. Participants could repeat this assessment at any time. Progress and difficulty were determined by the Valedo Home app.
- Modifications: to improve the attractivity of the study and recruitment, starting September 2019, the participants in the control group could borrow the Valedo Home and tablet for 3 weeks after completion of T4.
- Adherence measures: the exercises performed by the participants were automatically recorded on the tablet.
- Actual adherence: reported in the *Results* section.

### Data Preparation and Statistical Analysis

Data preparation and analysis were conducted in MATLAB R2018a (The MathWorks, Inc) and R (version 4.0.4; R Foundation for Statistical Computing). The simultaneously recorded data of the force platform and the IMUs were time-synchronized based on aligning a sideways leaning movement of the participants, shifting their weight to the left and the right, which was performed before and after each repetition. For this time synchronization, the movement had to be clearly distinct from the tasks and identifiable in both sources of data. This parallel recording was important for comparing force plate and IMU data. The beginning and the end of each balance and movement task were defined based on marker time stamps set in the IMU data during the assessment. The markers were inspected visually and corrected by hand before further analysis, as placement during the assessment was sometimes not optimal and occurred too early during the time-synchronization movement or too late during the task.

To assess the equivalence among the treatment groups at study entry (T1), participant characteristics were compared. Welch *t* test (2-tailed) or alternatively Wilcoxon rank-sum tests were used, if the data appeared to be not normally distributed based on Normal QQ plots or Shapiro-Wilk tests within groups. Dependent group *t* tests (2-tailed) were used to test whether change had occurred between T1 and T2, or if the assumptions were not met, Yuen tests, as provided by the R package WRS2 [64], were used. The hypotheses regarding the intervention effects were tested by comparing the change of the respective outcome (*Δ* outcome: T3-T2) between the intervention and the control group, predicting the more favorable outcome for the intervention group. These comparisons were performed each as intention-to-treat (ITT) and per-protocol (PP) analyses. In the ITT analyses, all participants who had been randomized at T2 were included. Missing values at T2 and T3 were replaced with the mean of the previous 2 assessments (T1 and T2) of the participant. For the PP analyses, participants who either had incomplete data or had been randomized to the intervention group but exercised <1 hour, between T2 and T3, were excluded. Comparisons were performed using independent group *t* tests (1-tailed), when the data were normally distributed according to Shapiro-Wilk tests, and a Levene test did not show heterogeneity of variances. Otherwise, Wilcoxon rank-sum tests were used. The a priori power analysis is reported within the study protocol [44]. We calculated post hoc power for the primary comparison using G*Power (Heinrich Heine Universität Düsseldorf) [65]. The effect size *r* observed for the ITT comparison of the primary outcome was converted to *d* using a web-based tool [66]. Power was calculated for a directed Wilcoxon rank-sum test with an *α* level set to .05 and the distribution menu set to *normal*.

Additional exploratory analyses to compare the absolute scores across all assessment visits including the second intervention period were conducted using mixed 2-way ANOVA. Only participants who completed the study (n=20) were included in these analyses. Missing data were replaced by mean scores of the previous assessments of that participant. Generalized *η*^2^ was used as effect size [48], and calculations were made using the r package rstatix (version 0.7.0; [50]). Shapiro-Wilk tests and Levene tests were used to test the assumptions of normality and homogeneity of variances. If the data did not fulfill the assumptions of normality and homogeneity of variances, different data transformations were explored. In cases where no suitable transformation was found, Friedman ANOVA was conducted across the assessment visits for each group separately, and group differences were compared at each assessment visit using the Bonferroni-corrected Wilcoxon rank-sum tests. Data on adherence were analyzed using descriptive statistics and graphs.

## Results

### Data Cleaning and Preparation

On the basis of visual inspection, orientation data from the IMU sensors were corrected in 2 cases where axes were flipped (15 trials of 2 participants). The data from a participant at T1 and another participant in the control group at T2 were discarded, because misplacement of the sensors was suspected. For a participant in the intervention group, no data for the T3 assessment were available, as the sensors had not been sufficiently charged. In the ITT analysis, all randomized participants were analyzed and missing values were replaced as described in the *Methods* section. For the ITT analysis of the balance and questionnaire data, 6 replacements (5 control and 1 intervention) were made for the T3 assessments. For the movement tasks, 1 replacement in the control group was made for the T2 assessments, and 7 replacements (5 control and 2 intervention) were made for the T3 assessments. For the PP analysis, participants for whom replacements had to be made were removed from the analysis. In addition, data from the participants in the intervention group (3 balance and questionnaires; 2 movement tasks), who had exercised <1 hour within the 3-week period and were excluded from the PP analysis, if this was not already the case when the participant was also a dropout, or the data had already been removed owing to insufficiently charged sensors.

During data analysis, it was discovered that some of the items of the WHOQOL-Bref at T3 in the German version had been collected with response options ranging from 1 to 4 instead of 1 to 5. Data collected with the affected items (items 3-9) were discarded for all assessment visits, and the scores of the scales were calculated without those items.

As not all data fulfilled the requirements for the 2-way mixed ANOVA for the analysis across all 4 assessment visits, the data were transformed where necessary. Transformations applied are reported in Table 1.

**Table 1:** Transformations applied to satisfy requirements for the analysis including all assessment visits in the two-way mixed ANOVA.

Outcome	Transformation
Mean anterior-posterior displacement	max(1 / (x + 1)) – (1 / (x + 1))
Mean medio-lateral displacement	log(x + 1)
Mean global displacement	log(x + 1)
Mean anterior-posterior velocity	None necessary
Mean medio-lateral velocity	max(1 / (x + 1)) – (1 / (x + 1))
Mean global velocity	None necessary
Box lift lumbar spine	log((x / 5) + 1)
Box lift hip	log((x / 5) + 1)
Waiter bow lumbar spine	log((x / 5) + 1)
Waiter bow hip	log((x / 5) + 1)
Pain intensity numeric rating scale	log(x + 1)
Roland Morris disability questionnaire	No suitable found
Quality of life physical subscale	log(x + 1)
Tampa Scale for Kinesiophobia, 11-item version	None necessary

### Duration Between Assessment Visits

The effective duration between T1 and T2 had a median of 21 days (IQR 5; minimum 17, maximum 97); between T2 and T3, 23 days (IQR 3; minimum 19, maximum 36); and between T3 and T4, 44 days (IQR 7.75; minimum 38, maximum 112). For a participant, the time span between T1 and T2 was extended to 97 days, and for 2 participants, the period between T3 and T4 was extended to 112 and 99 days, respectively, because of an interruption in the study owing to the COVID-19 pandemic. The period between T2 and T3 was not affected by extensions due to the COVID-19 pandemic.

### Participants and Baseline Characteristics

**Figure 3:**
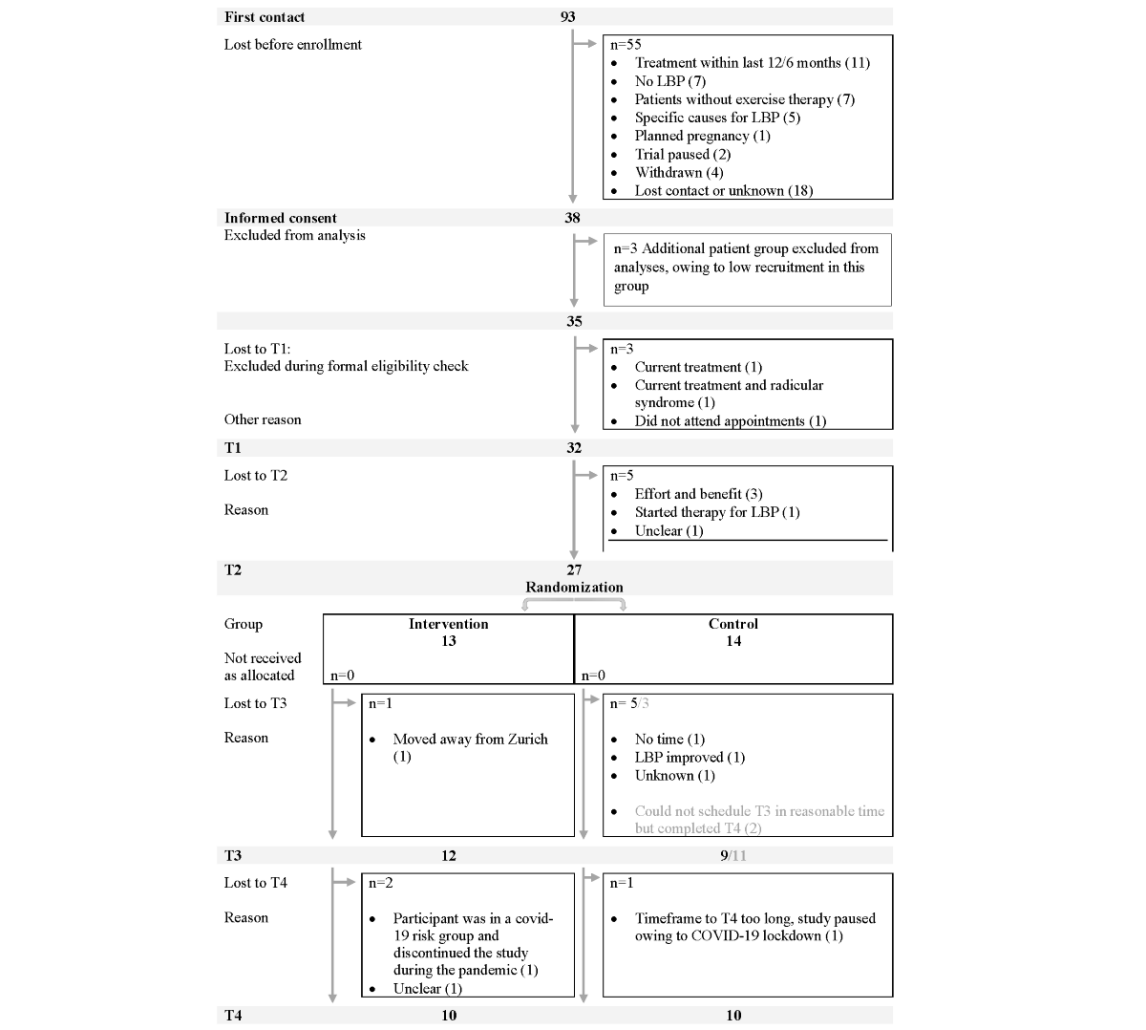
Participant flow through the study. Numbers of analyzed participants are reported in the text. LBP: low back pain.

As presented in Figure 3, a total of 93 participants made an initial contact and requested information regarding the study. Of those 93 participants, 38 (41%) provided written informed consent. At T1, a total of 32 participants, without recent treatment for LBP, were eligible for the study. In all, of 32 participants, 5 (16%) dropped out before randomization at T2 (n=27, 84%). Tables 2 and 3 show the participant characteristics at baseline. Between the participants randomized to the intervention and the control group, there were no significant differences in any outcome measure at T1 or T2, but analyses of change between T1 and T2 revealed that there was a significant reduction in pain intensity across participants who had been randomized (T1: median 3.00; mean 3.26, SD 1.56 and T2: median 2.00; mean 2.59, SD 1.34). Descriptive statistics on all outcomes at all assessment visits are reported in Table S1 in Multimedia Appendix 4 and comparisons of outcomes at T1 and T2 are shown in Table S2 in Multimedia Appendix 4.

**Table 2:** Participant characteristics at T1.

Characteristics	Control (n=14)		Intervention (n=13)		Comparison
	Median (range)	Mean (SD)	Median (range)	Mean (SD)	*P* value
Age (years)	37.50 (38.00)	40.14 (12.38)	34.00 (40.00)	40.85 (15.15)	.92^a^
Height (cm)	174.25 (36.00)	173.27 (8.61)	170.50 (18.50)	170.73 (6.55)	.39
Weight (kg)	73.55 (42.10)	76.01 (11.97)	74.10 (36.40)	72.56 (9.57)	.41
Age at first time LBP^b^ (years)	24.50 (33.00)	26.50 (10.51)	20.00 (29.00)	24.00 (9.52)	.52
LBP previous month (days)	10.00 (18.00)	9.43 (6.16)	11.00 (28.00)	5.46 (10.08)	.16^a^
Average pain intensity (0-10)	4.00 (5.00)	4.07 (1.33)	4.00 (4.00)	3.85 (1.07)	.63

^a^Wilcoxon rank-sum test.

^b^LBP: low back pain.

**Table 3:** Gender and language data of participants at T1.

Characteristic			Values
**Gender (female/male)**			
	Control	9/5	
	Intervention	8/5	
**Language (German/English)**			
	Control	11/3	
	Intervention	11/2	

### Change in Outcomes During the Intervention Period With Predefined Schedule

#### Overview

The change in the outcome variables between (T3-T2) was compared between both groups, for all outcome variables. All comparisons were performed as ITT and PP analyses. ITT analyses were performed with 14 participants in the control and 13 participants in the intervention groups, as randomized. PP analyses were conducted with 9 participants in each group, except for the movement tasks, where data from only 8 participants were available in the control group. Descriptive statistics and T2 and T3 scores for the ITT and PP analysis are reported in Table S3 in Multimedia Appendix 4.

#### Postural Balance

The primary outcome, change between T2 and T3 in mean AP displacement, did not differ among groups in the ITT analysis (control: median 0.01, range 3.91; mean 0.32, SD 0.95 and intervention: median 0.18, range 2.76; mean 0.31, SD 0.77; comparison: *W*=99; *P*=.36; *r*=0.07) and neither in the PP analysis (control: median 0.03, range 3.91; mean 0.45, SD 1.17 and intervention: median 0.05, range 2.51; mean 0.17, SD 0.69; comparison: *t*_16_=0.64; *P*=.73; *r*=0.16). The post hoc power for detecting the small effect observed in the ITT analysis, *r*=0.07 (equivalent to *d*=0.14), of the primary outcome was 0.1. No group differences in the ITT or PP analyses were found for the other postural balance parameters explored (Table 4). Multimedia Appendix 5 shows a graph of the postural balance data as it was used for the ITT analysis.

**Table 4:** Directed group comparisons of change in the balance outcomes between T2 and T3.

Outcome and analysis		Control			Intervention			Comparison			
		Median (range)	Mean (SD)	Median (range)		Mean (SD)	*t* test (*df*)		*W*	*P* value	*r*
***Δ* mean medio-lateral displacement**											
	ITT^a^	–0.22 (3.64)	–0.36 (0.84)	–0.15 (1.72)		–0.36 (0.58)	N/A^b^		89	.55^c^	0.02
	PP^d^	–0.18 (3.64)	–0.37 (1.05)	–0.29 (1.72)		–0.47 (0.61)	0.27 (16)		N/A	.40	0.07
***Δ* mean global displacement**											
	ITT	–0.14 (4.23)	–0.56 (1.12)	–0.18 (2.35)		–0.46 (0.80)	N/A		83	.66^c^	0.07
	PP	–0.13 (4.23)	–0.68 (1.39)	–0.18 (2.32)		–0.39 (0.73)	N/A		39	.57^c^	0.03
***Δ* mean anterior-posterior velocity**											
	ITT	–0.21 (5.48)	–0.49 (1.26)	–0.41 (4.32)		–0.83 (1.29)	0.70 (25)		N/A	.25	0.14
	PP	–0.66 (5.48)	–0.76 (1.53)	–0.22 (3.38)		–0.87 (1.17)	0.18 (16)		N/A	.43	0.05
***Δ* mean medio-lateral velocity**											
	ITT	–0.19 (2.37)	–0.33 (0.66)	–0.17 (4.12)		–0.23 (0.97)	–0.31 (25)		N/A	.62	0.06
	PP	–0.26 (2.37)	–0.38 (0.73)	–0.17 (1.53)		–0.26 (0.52)	–0.41 (16)		N/A	.66	0.10
***Δ* mean global velocity**											
	ITT	–0.48 (6.39)	–0.65 (1.50)	–0.29 (5.99)		–0.92 (1.73)	0.44 (25)		N/A	.33	0.09
	PP	–0.78 (6.39)	–0.93 (1.81)	–0.23 (3.64)		–0.98 (1.35)	0.06 (16)		N/A	.47	0.02

^a^ITT: intention-to-treat.

^b^N/A: not applicable.

^c^Wilcoxon rank-sum test.

^d^PP: per-protocol.

#### Movement Tasks

**Figure 4:**
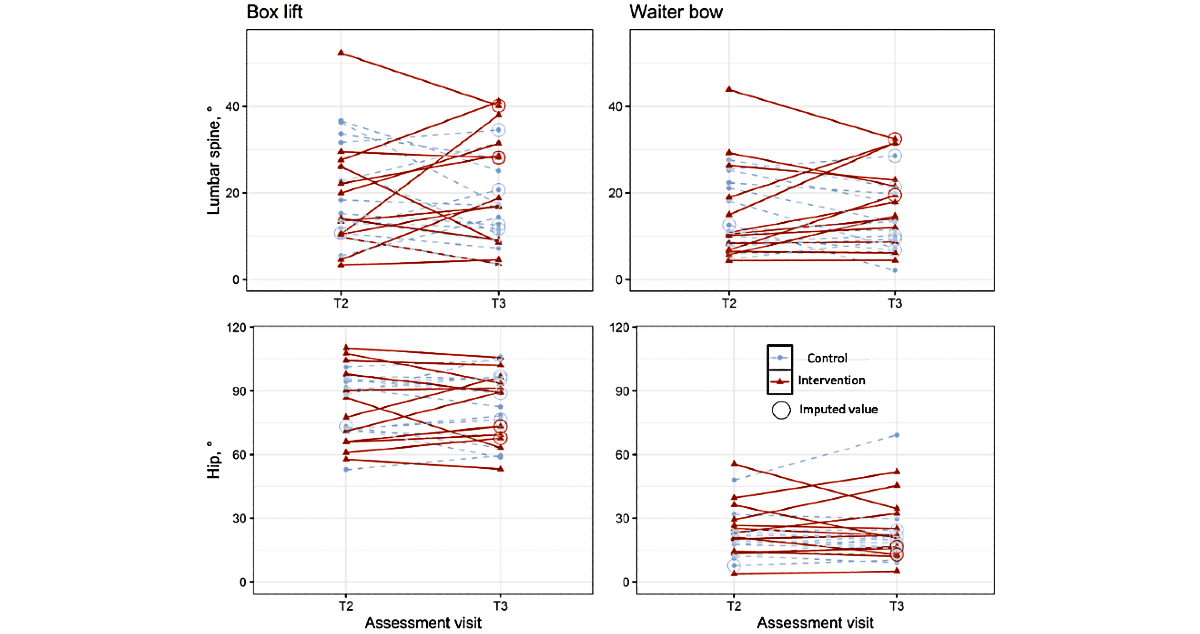
Lumbar spine and hip movement in degrees during the box lift and waiter bow task at T2 and T3. Data as included in the intention-to-treat analysis (control: n=14 and intervention n=13). Red triangles and solid lines show data of participants in the intervention group. Blue points and dashed lines show data of participants in the control group. Circled values represent imputed data.

Comparisons of change between T2 and T3 in lumbar and hip movement during the movement tasks are shown in Table 5 and Figure 4. There was no significant difference in either the ITT or the PP comparisons in accordance with our hypotheses. However, for the lumbar spine there were small decreases in the deviation from the starting position during task performance in the control group and small increases in the intervention group. Thus, the results descriptively showed a trend opposing our predictions with respect to the lumbar spine for both the box lift and waiter bow tasks with moderate effect sizes.

**Table 5:** Directed group comparisons of change in the movement tasks between T2 and T3.

Outcome and analysis		Control			Intervention			Comparison			
		Median (range)	Mean (SD)	Median (range)		Mean (SD)	*t* test (*df*)		*W*	*P* value	*r*
***Δ* box lift lumbar spine**											
	ITT^a^	–3.05 (27.86)	–3.00 (8.61)	3.37 (45.08)		3.25 (12.10)	–1.56 (25)		N/A^b^	.93	0.30
	PP^c^	–5.37 (27.80)	–5.05 (10.31)	6.69 (45.08)		6.03 (13.00)	–1.93 (15)		N/A	.96	0.45
***Δ* box lift hip**											
	ITT	0.31 (31.94)	–0.14 (8.52)	1.10 (42.64)		0.43 (12.04)	–0.14 (25)		N/A	.44	0.03
	PP	2.48 (31.94)	0.84 (10.21)	–2.27 (42.09)		–2.07 (12.11)	–0.53 (15)		N/A	.70	0.14
***Δ* waiter bow lumbar spine**											
	ITT	–1.12 (15.20)	–2.50 (5.22)	1.91 (28.04)		3.16 (8.22)	–2.15 (25)		N/A	.98	0.40
	PP	–1.12 (15.20)	–2.62 (5.51)	1.91 (24.28)		3.07 (7.14)	–1.82 (15)		N/A	.96	0.42
***Δ* waiter bow hip**											
	ITT	–0.85 (25.43)	1.50 (6.83)	1.32 (37.10)		–0.48 (10.33)	N/A		92	.53^d^	0.01
	PP	–1.81 (25.43)	2.46 (8.93)	1.32 (27.99)		0.41 (7.94)	N/A		33	.41^d^	0.07

^a^ITT: intention-to-treat.

^b^N/A: not applicable.

^c^PP: per-protocol.

^d^Wilcoxon rank-sum test.

#### Participant-Reported Outcomes

**Figure 5:**
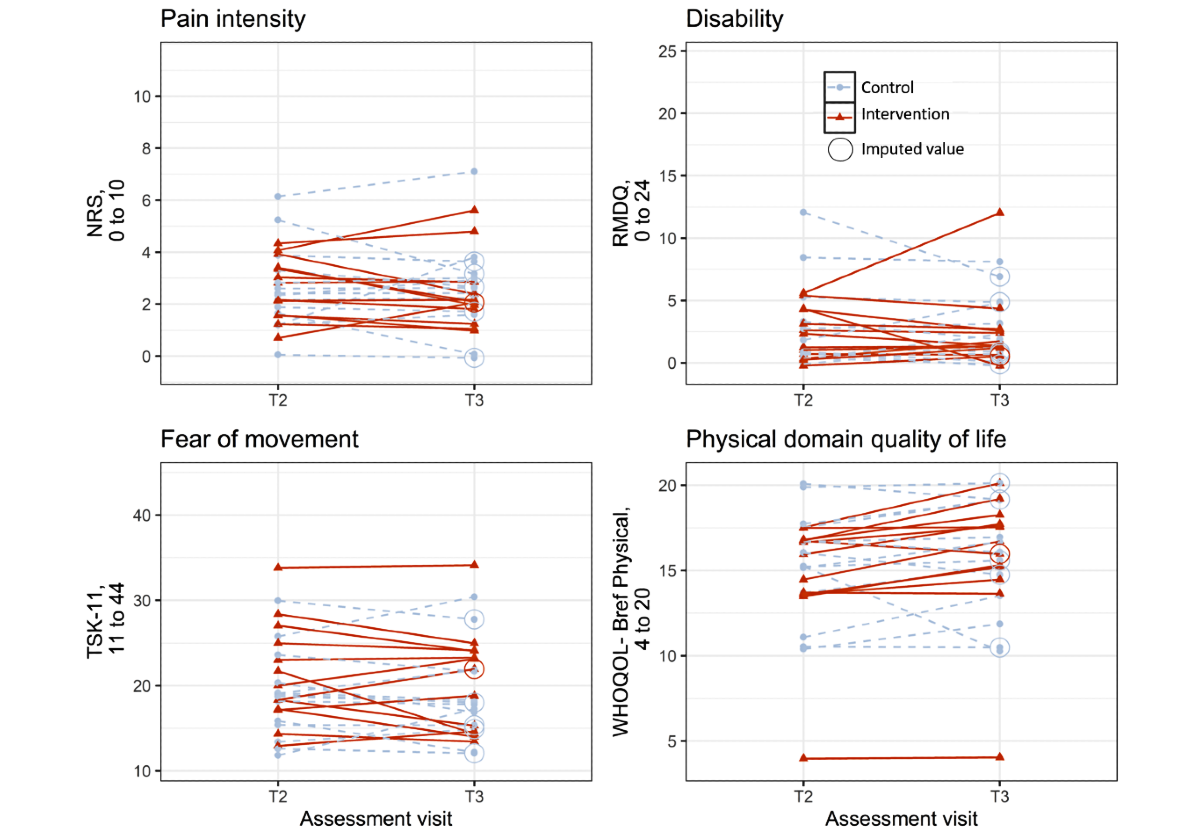
Scores of participant-reported outcomes for the assessment visits T2 and T3. Data as included in the intention-to-treat analysis (control: n=14 and intervention: n=13) are displayed. To represent all data in the graph despite exact overlap, small random values were added for the graphical representation of the data. Red triangles and solid lines show data of participants in the intervention group. Blue points and dashed lines show data of participants in the control group. Circled values represent imputed data. NRS: numeric rating scale; RMDQ: Roland Morris disability questionnaire; TSK-11: Tampa Scale for Kinesiophobia, 11-item version; WHOQOL-Bref Physical: Physical domain World Health Organization Quality of Life Questionnaire-short version.

The groups did not significantly differ in the change of scores in participant-reported outcomes in the ITT or PP analysis (Table 6 and Figure 5).

**Table 6:** Directed group comparisons of change in the participant-reported outcomes between T2 and T3.

Outcome and analysis		Control			Intervention			Comparison			
		Median (range)	Mean (SD)	Median (range)		Mean (SD)	*t* test (*df*)		*W*	*P* value	*r*
***Δ* pain intensity numeric rating scale**											
	ITT^a^	0.00 (5.00)	0.14 (1.18)	0.00 (4.00)		–0.12 (1.12)	0.58 (25)		N/A^b^	.28	0.12
	PP^c^	0.00 (5.00)	0.44 (1.33)	0.00 (3.00)		–0.44 (0.88)	1.67 (16)		N/A	.06	0.38
***Δ* Roland Morris disability questionnaire**											
	ITT	0.00 (8.50)	–0.25 (1.90)	0.00 (10.00)		0.12 (2.26)	N/A		88	.57^d^	0.03
	PP	0.00 (4.00)	0.33 (1.41)	–1.00 (6.00)		–0.55 (1.67)	1.22 (16)		N/A	.12	0.29
***Δ* Tampa Scale for Kinesiophobia, 11-item version**											
	ITT	–0.75 (9.00)	–0.04 (2.63)	–1.00 (11.50)		–0.88 (3.18)	0.76 (25)		N/A	.23	0.15
	PP	–1.00	0.11 (3.18)	–1.00		–1.22 (3.46)	0.85 (16)		N/A	.20	0.21
***Δ* quality of life physical subscale**											
	ITT	0.20 (7.20)	0.23 (1.83)	1.60 (3.20)		1.11 (1.06)	N/A		63.5	.09^d^	0.26
	PP	1.60 (7.20)	0.53 (2.23)	1.60 (2.40)		1.60 (0.80)	N/A		27.5	.13^d^	0.27
***Δ* quality of life psychological subscale**											
	ITT	0.00 (12.00)	–0.19 (2.65)	0.00 (4.00)		–2.56 (1.26)	N/A		101.5	.71^d^	0.10
	PP	1.33 (12.00)	–0.15 (3.36)	0.00 (4.00)		–0.44 (1.33)	N/A		50	.81^d^	0.20
***Δ* quality of life social subscale**											
	ITT	0.00 (5.33)	0.62 (1.35)	0.00 (6.67)		–0.15 (1.81)	N/A		114.5	.89^d^	0.23
	PP	0.00 (5.33)	0.89 (1.63)	0.00 (6.67)		0.30 (1.86)	0.72 (16)		N/A	.76	0.18
***Δ* quality of life environment subscale**											
	ITT	0.00 (5.33)	0.45 (1.32)	0.00 (4.00)		0.44 (1.58)	N/A		94	.57	0.03
	PP	0.00 (5.33)	0.67 (1.60)	0.00 (4.00)		0.22 (1.60)	N/A		50	.82^d^	0.20

^a^ITT: intention-to-treat.

^b^N/A: not applicable.

^c^PP: per-protocol.

^d^Wilcoxon rank-sum test.

### Exploratory Comparisons Across All Assessment Visits

Exploratory analyses were conducted across all 4 assessment visits among a subset of participants who remained in the study until T4 (n=20).

#### Postural Balance

There were no main effects of group or significant interaction effects (group by time) for any of the postural balance variables (Table 7). For AP velocity and global velocity, there was each a significant main effect of assessment visit, but none of the post hoc comparisons for the individual assessment visits showed significant differences. Descriptively displacement and velocity parameters increased between T1 and T2 and decreased from T2 to T3. This is surprising, as we did not expect to see such fluctuations in balance across time for the entire group of participants.

**Table 7:** Effects of Group and Assessment Visit on Postural Balance parameters within two-way mixed ANOVA.

Outcome	Group				Assessment visit				Group×assessment visit		
	*F* test (*df*)	*P* value	*η^2^_G_^a^*	*F* test (*df*)		*P* value	*η^2^_G_*	*F* test (*df*)		*P* value	*η^2^_G_*
Mean anterior-posterior displacement	0.25 (1,18)	.63	0.01	1.51 (3,54)		.22	0.02	1.21 (3,54)		.32	0.01
Mean medio-lateral displacement	1.21 (1,18)	.29	0.05	2.52 (3,54)		.07	0.03	0.10 (3,54)		.96	0.00
Mean global displacement	0.71 (1,18)	.41	0.03	2.59 (3,54)		.06	0.02	0.51 (3,54)		.68	0.01
Mean anterior-posterior velocity	1.60 (1,18)	.22	0.07	3.51 (3,54)		.02	0.03	0.28 (3,54)		.84	0.00
Mean medio-lateral velocity	0.03 (1,18)	.87	0.00	2.07 (3,54)		.12	0.01	0.21 (3,54)		.89	0.00
Mean global velocity	0.50 (1,18)	.49	0.02	3.61 (3,54)		.02	0.03	0.12 (3,54)		.95	0.00
Box lift lumbar spine	0.00 (1,18)	.99	0.00	1.32 (3,54)		.28	0.02	1.56 (3,54)		.21	0.03
Box lift hip	0.06 (1,18)	.81	0.00	0.27 (3,54)		.85	0.00	0.91 (3,54)		.44	0.01
Waiter bow lumbar spine	0.54 (1,18)	.47	0.02	0.22 (1.89,34.01)		.79^b^	0.00	0.82 (1.89,34.01)		.44^b^	0.02
Waiter bow hip	0.15 (1,18)	.71	0.01	1.40 (3,54)		.24	0.02	0.42 (3,54)		.74	0.01
Pain intensity numeric rating scale	0.94 (1,18)	.35	0.02	1.34 (3,54)		.27	0.04	0.76 (3,54)		.52	0.02
Quality of life physical subscale	0.26 (1,18)	.62	0.01	5.55 (1.78,31.98)		.01^b^	0.04	1.08 (1.78,31.98)		.34^b^	0.01
Tampa Scale for Kinesiophobia, 11-item version	0.22 (1,18)	.64	0.01	1.05 (3,54)		.38	0.01	0.14 (3,54)		.93	0.00

^a^Generalized *η^2^*.

^b^Greenhouse Geisser corrected.

#### Movement Tasks

There was no significant effect for group, assessment visit, and the interaction for lumbar spine or hip during the waiter bow and box lift tasks (Table 7).

#### Participant-Reported Outcomes

For the pain intensity NRS and fear of movement questionnaire, no significant effects for group, assessment visit, or their interaction were present (Table 7).

For the RMDQ scores, Friedman tests did not show significant differences across visits in the control group (*χ*^2^_3_=4.1; *P*=.25) or the intervention group (*χ*^2^_3_=6.0; *P*=.11). Bonferroni-corrected Wilcoxon rank-sum tests showed no difference among the groups at any assessment visit.

However, for the physical QOL, there was a significant main effect of assessment visit. Post hoc comparisons between assessment visits across both groups revealed that T3 scores were significantly higher than those at T2 (*t*_19_=3.71; *P*=.009). Results for the social, psychological, and environmental QOLs are reported in Multimedia Appendix 6.

### Adherence

**Figure 6:**
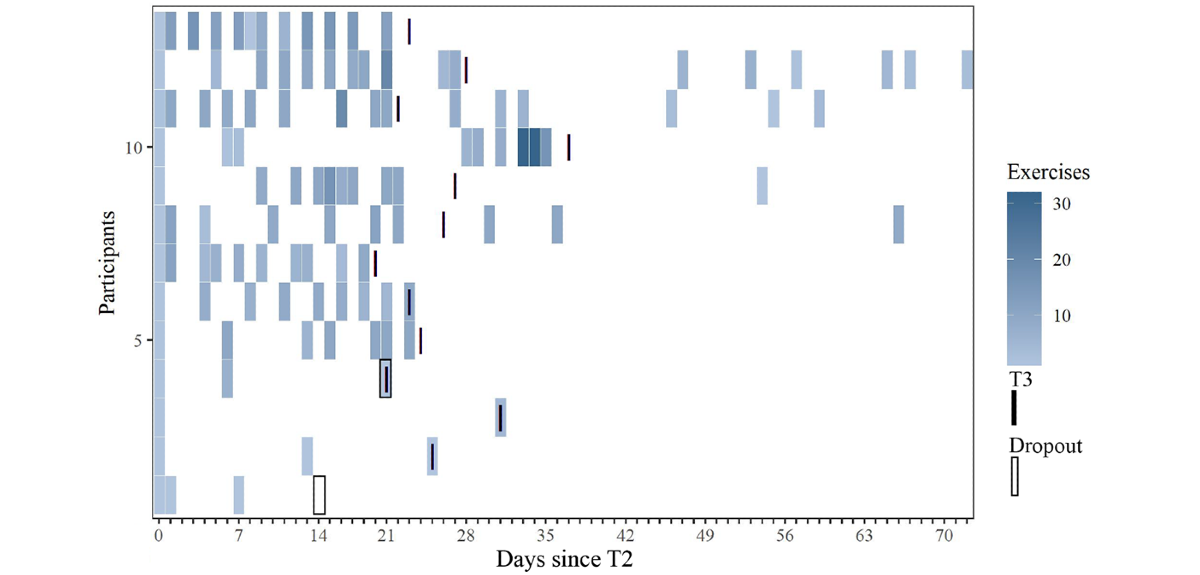
Exercises that have been completed during the study per participant per day. Darker color indicates a larger number of exercises performed. All exercises, including exercises that have not been intended as a part of the schedule are displayed. Black bars show assessment visit T3 and black boxes show dropouts at or before T3.

Participants in the intervention group were instructed to complete a fixed set of 90 exercises between the assessments T2 and T3. Of these exercises, a median of 61% (55/90; range 2%-99%) were completed. As not all exercises were performed with the specified duration and frequency, and some participants performed the exercises that were provided from the device but were not intended as part of the program, effective time spent exercising differed from the completion of the program. In this period with a predefined schedule (T2-T3), participants exercised a median of 77.2% (139/180; range 3%-202%) of the targeted exercising duration of 180 minutes. The exercising time of 4 participants exceeded 180 minutes. During the intervention period with a schedule, a total of 7 participants performed a median of 9 exercises (minimum 1, maximum 41), equivalent to 17 minutes (minimum 2, maximum 109) that were not part of the program. In the intervention period without a schedule, of the 11 participants who had remained in the study, 4 (36%) participants performed a median of 27 exercises (minimum 1, maximum 29), equivalent to 82 minutes (minimum 2, maximum 101). An overview of the number of any exercise performed is provided in Figure 6.

### Unintended Effects

There were no unintended effects that were related to the intervention. Although reasons for not adhering to the protocol were not assessed systematically and participants had been encouraged to contact the investigators with any difficulties, some participants in the intervention group reported problems with handling the devices. This included difficulties such as finding the right icon on the tablet and difficulties with the calibration of the IMUs and program failures of the tablet. These issues likely contributed to the low adherence of some participants.

## Discussion

### Principal Findings

Self-directed home exercising with feedback on trunk movements for a period of approximately 3 weeks did not enhance postural balance during quiet standing in study participants with LBP or significantly affect any other of the investigated outcomes. Comparisons of the groups with respect to the movement tasks showed, descriptively, a tendency toward slightly increased motion of the lumbar spine during both tasks in the intervention group, combined with a small reduction in the control group, which contradicted our predictions. Adherence to the scheduled exercising program was low. After the participants were no longer provided with a schedule to complete, only some participants kept using the training device repeatedly without specific instructions. Despite not showing intervention effects in this trial, it cannot be excluded that these interventions may still be beneficial when integrated into a therapy setting with patients. Furthermore, for other exercising interventions, it has been demonstrated that exercising could have more pronounced effects in patients than in other study participants with LBP [3]. A review showed that the results were positive for exercising with digital systems for LBP, when these exercises were delivered together with another intervention, but otherwise not [32].

### Comparison With Previous Work

#### Postural Balance

In this study investigating an exercise intervention using mobile sensors under self-directed home conditions in people with moderate LBP, no improvement of postural balance during quiet standing was found. To our knowledge, no other studies using feedback on trunk movements and similar assessments of postural balance have been conducted with participants with LBP. In a study where exergaming with the Nintendo Wii was included into the treatment, participants were not able to maintain single-legged stance for longer than before the intervention [67]. In contrast, in a study with older participants with diverse chronic musculoskeletal complaints and an exergame that mainly focused on translations of the body weight, several postural balance parameters improved but not relative to participants who had performed similar exercises without gamification [68]. A meta-analysis on studies with older participants without complaints suggests that exergames affect different measures of postural balance positively, but an enhancement of postural balance assessed under stable, unperturbed conditions could not be confirmed either [69]. Consistent with these observations, differences observable at the level of the trunk may not necessarily translate to changes in COP-based assessments during quiet standing [70]. Postural balance regulation is the product of the complex interaction of different structures and systems, with the capacity to adapt to changing conditions [71]. Thus, adaptations in balance should be explored additionally under varying assessment conditions. For example, assessments of trunk balance during sitting may provide an isolated assessment of trunk control [7] and could possibly reveal more subtle changes. Unexpectedly, we observed changes in some postural balance parameters across assessment visits, but statistically significant differences between individual visits were not found, which may be owing to a lack of power. The descriptive pattern did not indicate a continuous trend that could have been interpreted as learning or other effects of repetition.

#### Movement Tasks

In this study, the average amount of lumbar spine movement observed was comparable with the values reported earlier by other researchers [46]. However, contrary to our expectations, descriptively, the participants in the intervention group showed small increases in movement in the lumbar region during the movement tasks compared with the control group, who showed comparable reductions. This was the case despite the instructions to not bend or extend the lumbar spine during the assessment. Nevertheless, if only the increase in lumbar spine motion in the intervention group independently of the decrease in the control group is considered, this increase was only during the box lift task in the PP analysis (6.03°) slightly larger than the minimally detectable change value of 5.3°, which was described in the study our assessments were adapted from, by Matheve et al [46]. These descriptive observations may indicate that the intervention might rather impact mobility than the precise control of the lumbar spine. This interpretation would be in line with the finding of other investigators who found an expansion in ROM after a similar intervention but did not clearly state whether there was a difference in comparison with the group without the exercises [9]. No impact on an intervention on ROM was found in another study [10]. A recent meta-analysis challenged the assumption that people with LBP tend to bend their spine more in lift tasks [72], and restrictions in ROM in the lumbar region of people with LBP have already been described [14]. Furthermore, it was found that during a box lift task, participants with chronic LBP moved less in the lumbar region than participants without LBP [73]. Hence, an increase in movement in the lumbar spine would not necessarily constitute an undesirable outcome. Future studies should clarify the role of lumbar spine posture during lifting and the influence of exercising interventions on lifting behavior.

#### Participant-Reported Outcomes

There were no statistically significant differences between the change scores of groups in participant-reported outcomes. In contrast, some other studies investigating similar interventions found positive effects on pain assessments [9,10]. Nevertheless, it should be considered that pain NRSs could be error prone to some degree [74], and the power in this study may have been insufficient to detect an effect. A reduction in pain intensity across both groups was observed within the first 3 weeks of the study, where no intervention was provided. This effect could possibly be caused by participants initiating study participation during periods in which their pain was perceived as slightly worse than usual. The amount of pain appeared to be comparable with the value of approximately 2.5 obtained from visual analog scales, which had been reported in a review that revealed postural balance differences between people with and without LBP [17]*.* As we have observed, a small study found that exercises with postural feedback in addition to standard care was not superior in reducing disability than the usual treatment alone [11]. This is in contrast with the results of a different study, which indicated that disability could be improved [10]. However, in that specific study the feedback from the wearable device was not only provided during exercises but also during everyday activities [10]. In our study, the RMDQ mean scores were generally low, which may have limited the range of possible improvement. We did not find an intervention effect on physical QOL. Contrary to this result, in another study, an intervention effect on the physical subscale of the QOL measure short form-36 was observed [9]. A small trial using comparisons of the scores, before and following a similar intervention, showed significant improvements in pain, disability, and QOL [22]. We did not find an effect of the intervention on fear of movement, and neither an intervention effect was found in another study [10].

### Adherence

A particular strength of this work is the combination of an investigation of the exercising program at home in a period with a set training schedule and in a second interval, where participants could exercise as they wished. Comparison with studies on related interventions in home-based settings, which are considered similar, are difficult, as in a study a combined value including other exercises was investigated [11], and in another study, self-report methods had failed [6]. Furthermore, in a study that investigated exercising with the Nintendo Wii, completion of 71% of the advised time was achieved [36], which is comparable with the median of 77% obtained in this study. Nevertheless, the schedule provided was much more demanding and additional measures were used to improve adherence in the other study [36]. The comparatively short 3-week exercise period in combination with the low adherence may have limited the effects of the intervention. This assumption is in line with the findings of a review on virtual reality interventions, which indicated that interventions with more sessions may be more successful [33].

Results on adherence, considering time spent exercising, was more favorable than the number of exercises performed as requested by the investigators. Some participants exercised even more than required but did not follow the instructions precisely. In some cases, participants may have forgotten to reset the play time from the default 4 to 2 minutes, or the game may have motivated the participants to explore additional contents and may have provided stronger guidance than the instructions from the investigators. The 6-week period with flexible exercising opportunity resembled more closely to the conditions under which participants would be using such tools without the connection to a therapeutic setting. Only few participants kept exercising after T3. These results may imply that such interventions only get adopted by a small number of people or might rather be integrated within supervised programs on site. Within the setup of this study, it could not be determined whether the provided schedule or the participants’ commitment to comply with the study protocol resulted in higher amounts of exercises between T2 and T3. Future studies should investigate if and how automated scheduling options can help improve adherence and how they should be integrated. Although the Valedo app offers the option to generate an exercising plan, such functions could be placed more prominently. In addition, the context and the kind of assistance required should receive more attention. Recently, for example, blended therapy setups for people with LBP have been explored [75].

### Limitations

The low number of participants who could be recruited is an important limitation of this study. Although the individual components of the study protocol may not have been too time consuming, the overall effort associated with study participation, including diary methods and activity tracking not discussed in this manuscript, may have been a cause for low recruitment and retention rates. These assessments were included to answer additional research questions beyond the scope of a single manuscript but contributed to the effort by the study participants. In line with this presumption, reasons given for withdrawal were frequently related to time investment or perceived benefit and effort. This might also have contributed to the low adherence to the intervention. Time intervals between assessments were slightly stretched owing to frequent requests from participants to reschedule appointments, as the study participation was not part of an official treatment program and therefore often had to take place often outside of the working hours of the participants. The assessment of the movement tasks was preceded and followed by the participants shifting their weight to the sides and back, to time-synchronize the data from the IMUs with data collected simultaneously from the force platform. Although supporting analyses of change between T2 and T3, where data from trials that appeared to be performed from an unstable starting position were removed, appeared similar, this setup could have influenced the results. The study participants could not be blinded, and with most assessments conducted in the field, it could not be ruled out that the participants completed all exercises themselves. In a case with a particularly high number of exercises, it was suspected that other people may have completed some of those exercises. We did not record the use of pain medication during the study; therefore, confounding effects of pain medication could not be ruled out. Further, although we consider the availability of the questionnaires in different languages as a strength, this setup may have caused inconsistencies between the questionnaires that different participants received.

### Conclusions

The results obtained in this study indicate that exercising with feedback on trunk movements alone may not influence postural balance during quiet standing in people with only moderate LBP intensity and disability. No significant intervention effects on lumbar spine and hip movement, pain intensity, disability, QOL domains, and fear of movement were observed. These results must be seen within the context of a small sample size and low adherence to the intervention, resulting in low doses of exercise. More work in this field is required; for example, to establish the effect of interventions using feedback on trunk movements in people more severely affected by LBP and clarify more proximal effects on trunk movement properties. As the amount of exercising dropped substantially in the intervention period without a schedule, future studies should investigate the impact of different scheduling options and explore such interventions in combination with other therapeutic settings or other strategies to improve adherence.

## Appendix

Multimedia Appendix 1 CONSORT-EHEALTH (Consolidated Standards of Reporting Trials of Electronic and Mobile Health Applications and Online Telehealth; version 1.6.1) submission publication form.

Multimedia Appendix 2 Graphical representation of center of pressure parameters.

Multimedia Appendix 3 Video showing the exercises.

Multimedia Appendix 4 Descriptive statistics and baseline comparisons.

Multimedia Appendix 5 Data used for the intention-to-treat analysis of center of pressure data.

Multimedia Appendix 6 Additional comparisons including all assessment visits.

## Abbreviations

AP: anterior-posterior
CONSORT-EHEALTH: Consolidated Standards of Reporting Trials of Electronic and Mobile Health Applications and Online Telehealth
COP: center of pressure
IMU: inertial measurement unit
ITT: intention-to-treat
LBP: low back pain
NRS: numeric rating scale
PP: per-protocol
QOL: quality of life
REDCap: Research Electronic Data Capture
RMDQ: Roland Morris disability questionnaire
ROM: range of motion
TIDieR: Template for Intervention Description and Replication
WHOQOL-Bref: World Health Organization Quality of Life Questionnaire-short version
