# Supplementary material for: Feedback on Trunk Movements From an Electronic Game to Improve Postural Balance in People With Nonspecific Low Back Pain: Pilot Randomized Controlled Trial
Source: JMIR Serious Games. 2022 Jun 10;10(2):e31685. doi: 10.2196/31685 (PMC9233263; doi:10.2196/31685)
Supplement: Multimedia Appendix 2 [file games_v10i2e31685_app2.pdf]

Multimedia Appendix 2: Graphical representation of center of pressure parameters.

Calculations were based on:

Prieto TE, Myklebust JB, Hoffmann RG, Lovett EG, Myklebust BM. Measures of postural steadiness: Differences between healthy young and elderly adults. IEEE Trans Biomed Eng. 1996 Sep;43(9):956–66. PMID:9214811

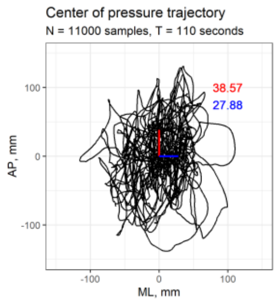

Figure S2.1: Example of a center of pressure recoding. The red value shows mean anterior-posterior displacement, blue shows mean medio-lateral displacement.

Displacements:

Describe the *average* distance of data points in the trajectory from the center:

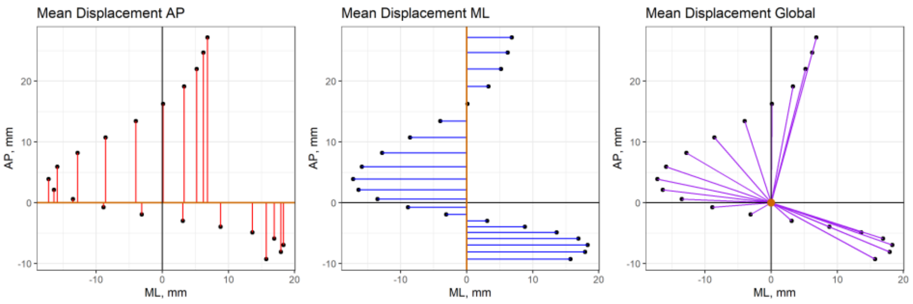

Given the data is centered to zero:

$$\text{Mean Displacement AP} = \frac{\sum_1^N |AP_n|}{N}$$

Velocities:

The path length traveled divided by time:

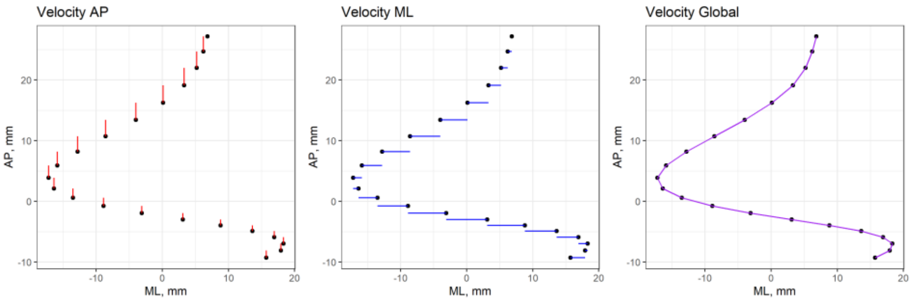

$$\text{Velocity AP} = \frac{\sum_1^{N-1}(|AP_{n+1} - AP_n|)}{t}$$
