# Supplementary material for: Feedback on Trunk Movements From an Electronic Game to Improve Postural Balance in People With Nonspecific Low Back Pain: Pilot Randomized Controlled Trial
Source: JMIR Serious Games. 2022 Jun 10;10(2):e31685. doi: 10.2196/31685 (PMC9233263; doi:10.2196/31685)
Supplement: Multimedia Appendix 4 [file games_v10i2e31685_app4.pdf]

Multimedia Appendix 4. Supplementary Tables.

Table S1: Descriptive statistics of outcome measures at each assessment visit

Outcome	Control median, mean (*SD*)	Intervention median, mean (*SD*)
**Assessment Visit T1**		
n	14	13
Displacement AP	4.27, 4.68 (1.59)	3.72, 3.97 (1.01)
Displacement ML	2.16, 2.46 (1.19)	1.97, 2.08 (0.80)
Displacement Global	5.26, 5.78 (2.10)	4.62, 4.89 (1.38)
Velocity AP	7.70, 8.62 (2.33)	9.96, 10.00 (2.78)
Velocity ML	3.54, 3.97 (1.66)	3.57, 3.99 (1.33)
Velocity Global	9.69, 10.31 (2.92)	11.19, 11.54 (3.10)
Lumbar Spine Box Lifting	17.46, 19.68 (11.55)	14.20, 15.68 (9.19)
Hip Box Lifting	83.51, 85.13 (16.58)	80.57, 84.42 (17.49)
Lumbar Spine Waiter Bow	12.43, 14.32 (8.77)	13.26, 15.10 (8.89)
Hip Waiter Bow	16.41, 21.06 (12.14)	18.86, 21.39 (13.45)
NRS	3.00, 3.21 (1.85)	3.00, 3.31 (1.25)
RMDQ	1.50, 2.57 (2.85)	3.00, 3.15 (1.86)
QOL Physical	4.14, 4.11 (0.61)	4.14, 3.99 (0.75)
QOL Psychological	4.00, 3.92 (0.71)	4.00, 4.01 (0.58)
QOL Social	4.00, 3.95 (0.85)	4.00, 3.90 (0.64)
QOL Environment	4.06, 4.18 (0.55)	4.25, 4.25 (0.55)
TSK-11	18.00, 18.79 (4.61)	22.00, 20.85 (4.45)
**Assessment Visit T2**		
n	14 (13 Movement tasks)	13
Displacement AP	4.86, 4.72 (1.65)	3.94, 4.41 (1.34)
Displacement ML	2.39, 2.80 (1.52)	2.47, 2.41 (1.06)
Displacement Global	5.40, 6.07 (2.22)	4.89, 5.46 (1.74)
Velocity AP	8.88, 8.84 (2.38)	9.87, 10.17 (1.92)
Velocity ML	3.68, 4.10 (1.91)	3.77, 4.02 (1.26)
Velocity Global	10.15, 10.59 (3.15)	11.43, 11.74 (2.11)
Lumbar Spine Box Lifting	18.39, 21.12 (10.73)	14.07, 18.75 (13.21)
Hip Box Lifting	90.27, 84.19 (14.45)	77.57, 81.72 (18.78)
Lumbar Spine Waiter Bow	11.11, 15.30 (8.23)	10.47, 15.11 (11.64)
Hip Waiter Bow	19.62, 21.79 (9.58)	23.26, 24.78 (13.41)
NRS	2.00, 2.57 (1.60)	3.00, 2.62 (1.04)
RMDQ	1.00, 2.71 (3.47)	2.00, 2.31 (2.02)
QOL Physical	4.21, 4.02 (0.74)	3.86, 3.92 (0.75)
QOL Psychological	4.00, 3.88 (0.76)	4.00, 3.79 (0.89)
QOL Social	3.83, 3.90 (0.88)	4.00, 3.85 (0.90)
QOL Environment	4.19, 4.06 (0.68)	4.00, 3.96 (1.05)
TSK-11	19.00, 18.79 (5.13)	20.00, 21.23 (6.04)
**Assessment Visit T3**		
n	9	12 (11 Motor control tasks)
Displacement AP	3.90, 4.18 (0.83)	3.86, 4.13 (1.08)
Displacement ML	1.73, 2.21 (0.92)	1.89, 2.05 (0.89)
Displacement Global	5.03, 5.16 (1.16)	4.67, 5.03 (1.31)
Velocity AP	8.15, 8.02 (2.37)	9.40, 9.52 (2.68)
Velocity ML	3.32, 3.63 (1.35)	3.29, 3.86 (1.49)
Velocity Global	9.36, 9.53 (2.82)	10.88, 11.02 (3.11)
Lumbar Spine Box Lifting	14.36, 15.07 (8.89)	17.10, 19.79 (13.23)
Hip Box Lifting	82.52, 82.07 (18.60)	89.46, 84.28 (16.94)

Lumbar Spine Waiter Bow	11.51, 11.16 (5.62)	14.70, 16.88 (9.30)
Hip Waiter Bow	20.08, 24.27 (18.00)	21.96, 26.04 (13.97)
NRS	3.00, 2.88 (1.90)	2.00, 2.50 (1.57)
RMDQ	2.00, 2.44 (2.60)	1.50, 2.58 (3.18)
QOL Physical	4.14, 4.11 (0.61)	4.43, 4.05 (1.08)
QOL Psychological	3.83, 3.63 (0.78)	4.06, 3.94 (1.01)
QOL Social	4.00, 3.96 (0.54)	4.00, 3.86 (1.06)
QOL Environment	4.25, 4.29 (0.32)	4.38, 4.21 (0.96)
TSK-11	18.00, 19.33 (4.97)	21.00, 20.25 (6.34)
**Assessment Visit T4**		
n	10	10
Displacement AP	4.44, 4.51 (1.67)	3.66, 3.78 (1.01)
Displacement ML	2.54, 2.59 (1.22)	2.02, 2.16 (1.09)
Displacement Global	5.51, 5.69 (2.16)	4.73, 4.76 (1.56)
Velocity AP	7.88, 8.33 (2.22)	9.09, 9.17 (1.76)
Velocity ML	3.35, 3.99 (1.83)	3.74, 3.79 (1.27)
Velocity Global	9.66, 10.05 (2.96)	11.17, 10.68 (1.90)
Lumbar Spine Box Lifting	15.01, 18.67 (12.57)	24.45, 23.66 (14.49)
Hip Box Lifting	86.09, 85.70 (14.10)	83.01, 79.51 (21.53)
Lumbar Spine Waiter Bow	13.13, 13.46 (8.55)	18.76, 17.46 (9.18)
Hip Waiter Bow	20.85, 22.35 (8.72)	23.67, 25.05 (14.77)
NRS	3.00, 3.20 (1.03)	2.00, 2.30 (1.49)
RMDQ	2.50, 3.40 (2.95)	1.50, 2.10 (2.51)
QOL Physical	4.00, 3.96 (0.84)	4.36, 4.01 (0.92)
QOL Psychological	4.17, 3.92 (0.78)	3.92, 3.92 (0.69)
QOL Social	4.00, 3.80 (0.82)	4.00, 3.73 (1.12)
QOL Environment	4.06, 4.05 (0.71)	4.50, 4.30 (0.60)
TSK-11	20.50, 20.10 (4.84)	17.00, 18.50 (4.90)

Table S2. Comparison of outcomes of the randomized sample at T1 and T2 and between T1 and T2.

	T1		T2		T1 vs. T2	
Outcome	*t(df)/ W*	*P*	*t(df)/ W*	*P*	*t(df)/* $t_y(df)$	*P*
Displacement AP	120	.17[a]	0.77(23.28)	.45	−1.17(16)	.26[b]
Displacement ML	110	.38[a]	95	.87[a]	−1.77 (26)	.09
Displacement Global	116	.24[a]	0.79(24.37)	.44	−1.77 (26)	.09
Velocity AP	−1.38 (23.53)	.18	−1.60(24.57)	.12	−0.64(26)	.53
Velocity ML	83	.72[a]	84	.76[a]	−0.43(26)	.67
Velocity Global	−1.06 (24.55)	.30	−1.12(22.83)	.28	−0.64(26)	.53
LS Box Lifting	1.00 (24.46)	.33	101	.42[a]	−0.89(25)	.38
H Box Lifting	0.11 (24.58)	.91	95	.61[a]	1.01(25)	.32
LS Waiter's Bow	−0.23 (24.80)	.82	93	.69[a]	0.21(15)	.83[b]
H Waiter's Bow	83	.72[a]	69	.45[a]	−1.18(25)	.25
NRS	−0.15 (22.95)	.88	−0.08(22.50)	.93	2.11(26)	.045
RMDQ	67.5	.26[a]	87	.86[a]	1.53(16)	.15[b]
QOL- Physical	94.5	.88[a]	98.5	.73[a]	1.15(16)	.27[b]
QOL- Psychological	0.03 (24.99)	.98	106.5	.46[a]	−0.25(16)	.81[b]
QOL- Social	101	.64[a]	0.17(24.77)	.87	0(16)	.99[b]
QOL- Environment	−0.23 (24.02)	.82	91.5	1.00[a]	0.13(16)	.90[b]
TSK-11	−1.18 (24.96)	.25	−1.13(23.66)	.27	−0.29(26)	.77

[a] Comparison Wilcoxon rank-sum test.

[b] Comparison Yuen Test.

Table S3: Descriptive statistics of data used for intention-to-treat and per-protocol analyses of T2 and T3.

	T2		T3	
Analysis ITT/PP	Control median, mean (*SD*)	Intervention median, mean (*SD*)	Control median, mean (*SD*)	Intervention median, mean (*SD*)
**Mean anterior-posterior displacement**				
ITT	4.86, 4.72 (1.64)	3.94, 4.41 (1.34)	4.21, 4.40 (1.06)	3.85, 4.10 (1.04)
PP	4.73, 4.64 (1.81)	3.77, 4.18 (1.34)	3.90, 4.18 (0.83)	3.57, 4.01 (1.23)
**Mean medio-lateral displacement**				
ITT	2.39, 2.80 (1.52)	2.47, 2.41 (1.06)	2.03, 2.44 (1.06)	1.90, 2.05 (0.85)
PP	1.87, 2.58 (1.54)	2.00, 2.11 (1.07)	1.73, 2.21 (0.92)	1.71, 1.63 (0.55)
**Mean global displacement**				
ITT	5.40, 6.07 (2.22)	4.89, 5.46 (1.74)	5.29, 5.51 (1.54)	4.75, 5.01 (1.26)
PP	5.08, 5.84 (2.28)	4.69, 5.06 (1.70)	5.03, 5.16 (1.16)	4.28, 4.66 (1.31)
**Mean anterior-posterior velocity**				
ITT	8.88, 8.84 (2.38)	9.87, 10.17 (1.92)	8.08, 8.36 (2.26)	9.08, 9.34 (2.64)
PP	9.36, 8.78 (2.59)	9.87, 10.22 (1.84)	8.15, 8.02 (2.37)	9.08, 9.34 (2.44)
**Mean medio-lateral velocity**				
ITT	3.68, 4.10 (1.91)	3.77, 4.02 (1.26)	3.19, 3.77 (1.65)	2.99, 3.79 (1.45)
PP	3.58, 4.01 (1.53)	3.59, 3.61 (1.00)	3.32, 3.63 (1.35)	2.86, 3.35 (1.09)
**Mean global velocity**				
ITT	10.15, 10.59 (3.15)	11.43, 11.74 (2.11)	9.62, 9.94 (2.85)	9.93, 10.82 (3.06)
PP	10.20, 10.45 (3.18)	11.15, 11.52 (1.94)	9.36, 9.53 (2.82)	9.93, 10.55 (2.66)
**Box lift lumbar spine**				
ITT	16.80, 20.38 (10.68)	14.07, 18.75 (13.21)	15.69, 17.38 (9.43)	18.79, 22.00 (13.45)
PP	20.56, 21.10 (11.56)	10.49, 13.97 (8.75)	15.69, 16.06 (8.96)	17.10, 20.00 (13.95)
**Box lift hip**				
ITT	89.26, 83.41 (14.19)	77.57, 81.72 (18.78)	85.72, 83.26 (16.19)	89.28, 82.16 (16.35)
PP	89.95, 83.61 (16.33)	86.84, 84.43 (20.04)	86.25, 84.44 (18.37)	89.46, 82.36 (18.23)
**Waiter bow lumbar spine**				
ITT	11.83, 15.10 (7.94)	10.47, 15.11 (11.64)	10.80, 12.61 (7.09)	17.86, 18.27 (9.53)
PP	14.61, 14.91 (7.81)	10.47, 13.38 (8.76)	12.40, 12.29 (4.78)	14.70, 16.44 (8.17)
**Waiter bow hip**				
ITT	19.49, 20.79 (9.94)	23.26, 24.78 (13.41)	19.81, 22.29 (14.58)	21.78, 24.30 (13.46)
PP	21.85, 23.55 (11.84)	23.26, 22.53 (11.23)	20.98, 26.00 (18.42)	21.78, 22.94 (13.31)
**Pain intensity numeric rating scale**				
ITT	2.00, 2.57 (1.60)	3.00, 2.62 (1.04)	3.00, 2.71 (1.76)	2.00, 2.50 (1.50)
PP	2.00, 2.44 (1.51)	3.00, 2.67 (1.00)	3.00, 2.89 (1.90)	2.00, 2.22 (1.30)
**Roland Morris disability questionnaire**				
ITT	1.00, 2.71 (3.47)	2.00, 2.31 (2.02)	1.50, 2.46 (2.58)	1.00, 2.42 (3.09)
PP	1.00, 2.11 (2.47)	2.00, 2.22 (1.86)	2.00, 2.44 (2.60)	1.00, 1.67 (1.22)
**Quality of life physical subscale**				
ITT	15.60, 15.43 (3.13)	16.00, 14.71 (3.60)	15.80, 15.66 (3.20)	16.80, 15.82 (4.02)
PP	15.20, 14.93 (2.68)	16.80, 15.91 (1.62)	16.00, 15.47 (3.02)	17.60, 17.51 (1.62)
**Quality of life psychological subscale**				
ITT	16.67, 15.71 (3.52)	16.00, 14.87 (3.88)	16.00, 15.52 (3.66)	14.67, 14.62 (3.76)
PP	17.33, 15.70 (2.81)	16.00, 16.30 (2.08)	16.00, 15.56 (3.20)	16.00, 15.85 (2.26)
**Quality of life social subscale**				
ITT	15.33, 15.62 (3.52)	16.00, 15.38 (3.59)	16.00, 16.24 (2.93)	16.00, 15.23 (4.13)
PP	14.67, 14.96 (2.96)	16.00, 16.89 (2.31)	16.00, 15.85 (2.15)	17.33, 17.19 (1.82)
**Quality of life environmental subscale**				
ITT	16.67, 16.48 (2.85)	16.00, 16.05 (4.45)	17.00, 16.93 (2.27)	18.00, 16.49 (3.79)
PP	16.00, 16.44 (2.36)	18.67, 18.07 (2.01)	16.67, 17.11 (1.29)	18.00, 18.30 (1.70)
**Tampa scale of kinesiophobia -11 item version**				
ITT	19.00, 18.79 (5.13)	20.00, 21.23 (6.04)	18.00, 18.75 (5.22)	21.50, 20.35 (6.08)
PP	19.00, 19.22 (4.09)	18.00, 19.11 (4.70)	18.00, 19.33 (4.97)	15.00, 17.89 (4.68)
