# Supplementary material for: Feedback on Trunk Movements From an Electronic Game to Improve Postural Balance in People With Nonspecific Low Back Pain: Pilot Randomized Controlled Trial
Source: JMIR Serious Games. 2022 Jun 10;10(2):e31685. doi: 10.2196/31685 (PMC9233263; doi:10.2196/31685)
Supplement: Multimedia Appendix 5 [file games_v10i2e31685_app5.pdf]

Multimedia Appendix 5: Figure showing data used for the ITT analysis of COP data

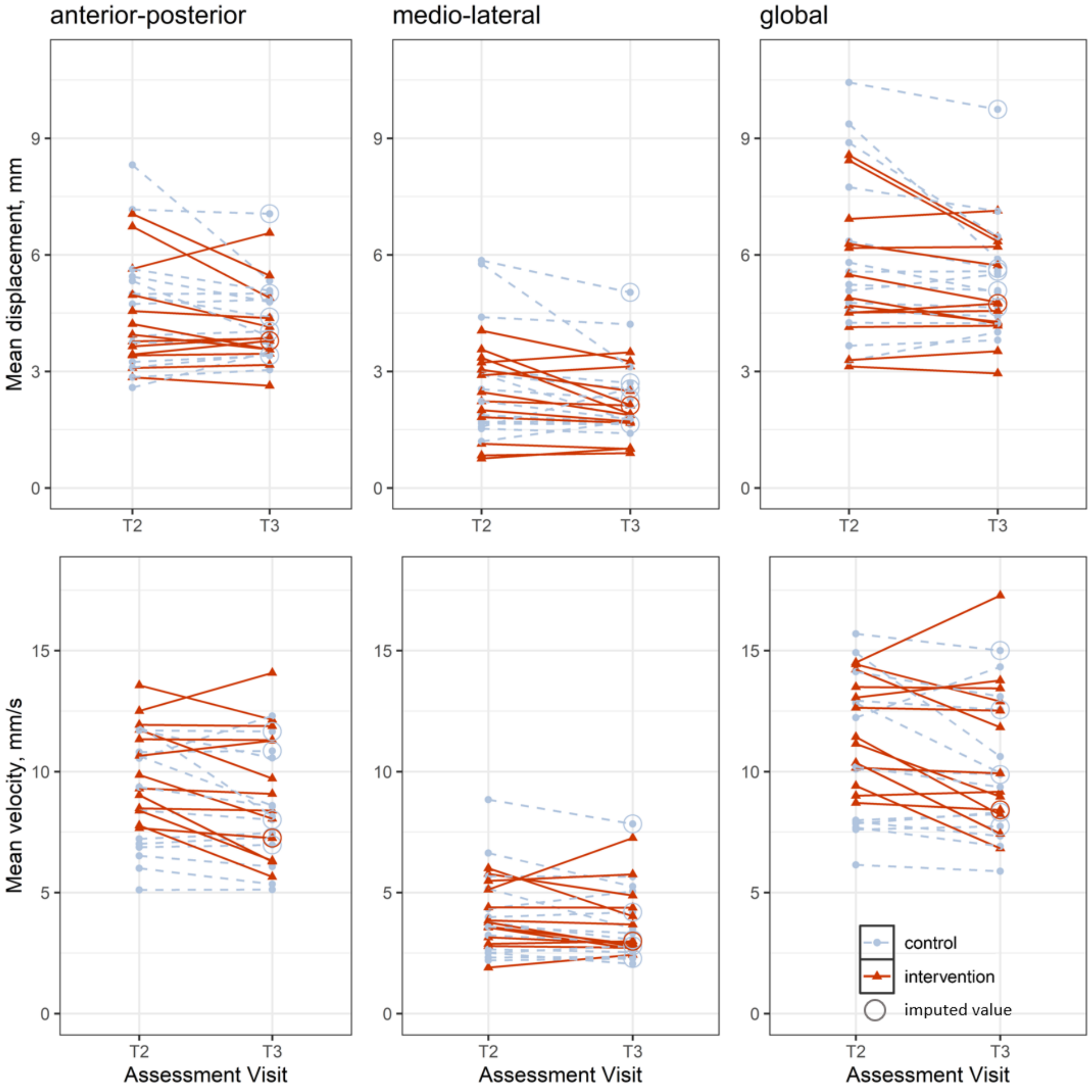

Figure S5. Postural balance parameters for the assessment visits T2 and T3. Data as included in the intention-to-treat analysis (control: n = 14, intervention n = 13) is displayed. Red triangles and solid lines show data of participants in the intervention group. Blue points and dashed lines show data of participants in the control group. Circled values represent imputed data.
