# Supplementary material for: Feedback on Trunk Movements From an Electronic Game to Improve Postural Balance in People With Nonspecific Low Back Pain: Pilot Randomized Controlled Trial
Source: JMIR Serious Games. 2022 Jun 10;10(2):e31685. doi: 10.2196/31685 (PMC9233263; doi:10.2196/31685)
Supplement: Multimedia Appendix 6 [file games_v10i2e31685_app6.pdf]

Multimedia Appendix 6. Additional Comparisons including all assessment visits.

QOL Psychological Effect

To meet the requirements of a two-way mixed ANOVA Psychological Quality of life scores were transformed as log((x/4)+1). There was no statistically significant effect of Group: $F_{1, 18}$=0.01; P=.93; η^2_G=0.00, Assessment Visit: $F_{3,54}$=0.87; P=.46; η^2_G=0.00, and their interaction: $F_{3,54}$=2.60; P=.06; η^2_G=0.01.

Social Quality of Life

No transformation was found for Social Quality of Life scores to fulfil the requirements of parametric analysis. There was no effect of Assessment visit on social quality of life in the control group χ^2_3=2.69; P=.44 and neither in the intervention group χ^2_3=5.76; P=.12 using Friedmann tests. None of the Bonferroni corrected Wilcoxon rank sum tests at each assessment visit showed statistically significant differences between the groups.

Environmental Quality of Life

Environmental Quality of Life could not be transformed to satisfy the assumptions of a two-way mixed ANOVA. Friedmann tests showed no effect of Assessment visit on social quality of life in the control group χ^2_3=2.12; P=.55 and neither in the intervention group χ^2_3=0.76; P=.86. None of the Bonferroni corrected Wilcoxon rank sum tests comparing groups at each assessment visit was statistically significant.
